# Advancements in Revascularization Strategies for Acute Mesenteric Ischemia: A Comprehensive Review

**DOI:** 10.3390/jcm13020570

**Published:** 2024-01-19

**Authors:** Jacob J. Gries, Hafeez Ul Hassan Virk, Bing Chen, Takashi Sakamoto, Mahboob Alam, Chayakrit Krittanawong

**Affiliations:** 1 Department of Internal Medicine, Geisinger Medical Center, Danville, PA 17822, USA; jgries1@geisinger.edu; 2 Harrington Heart & Vascular Institute, Case Western Reserve University, University Hospitals Cleveland Medical Center, Cleveland, OH 44106, USA; 3 Department of Gastroenterology and Hepatology, Geisinger Medical Center, Danville, PA 17822, USA; 4 Department of Gastroenterological Surgery, Gastroenterological Center, Cancer Institute Hospital of Japanese Foundation for Cancer Research, Tokyo 113-0033, Japan; 5 Department of Clinical Epidemiology & Health Economics, School of Public Health, The University of Tokyo, Tokyo 113-0033, Japan; 6 Section of Cardiology, Baylor College of Medicine, Houston, TX 77030, USA; 7 Cardiology Division, NYU Langone Health and NYU School of Medicine, New York, NY 10016, USA

**Keywords:** revascularization, acute mesenteric ischemia (AMI), endovascularization, hybrid

## Abstract

Even with modern advancements in the management of acute mesenteric ischemia over the past decade, morbidity and mortality remain high, and the best primary treatment modality is still debated amongst interventionalists. Traditionally, interventionalists have favored an open surgical approach but are now trending for endovascular interventions due to apparent reduced mortality and complications. Newer studies suggest hybrid approaches, and intestinal stroke centers may be superior to either strategy alone. This narrative review will explore the natural history of acute mesenteric ischemia with the aim of increasing interventionalist awareness of modern advancements in revascularization strategies for this devastating disease.

## 1. Introduction

Acute mesenteric ischemia (AMI) poses a formidable challenge in clinical practice. It demands an adept understanding of visceral anatomy, pathophysiology, and contemporary treatment strategies to quickly diagnose, treat, and mitigate its high mortality risk. Despite its relatively low prevalence rate of only 0.09% to 0.2% of all hospitalizations [1], the mortality rate of AMI can exceed 70% if left untreated [2]. Knowledge of the three different etiologies of acute mesenteric ischemia, including arterial thromboembolic, venous thrombosis, or nonocclusive types, can aid in the quick recognition and early initiation of therapy in these patients. 

### 1.1. Visceral Artery Anatomy and Function

The three major arteries that supply blood and nutrients to and remove waste from the gastrointestinal tract are the celiac artery, superior mesenteric artery, and inferior mesenteric artery [3,4]. The celiac artery supplies the stomach, proximal duodenum, and spleen [3,4]. The superior mesenteric artery supplies the region from the small intestine to the distal third of the transverse colon [3,4]. The inferior mesenteric artery is the smallest of the vessels and supplies the distal colon and proximal third of the rectum [3,4]. There is an extensive network of collateralization between these vessels to allow for compensatory blood flow should one vessel become compromised [3,4]. Because of this, ischemic events in two or more vessels are necessary to generate acute mesenteric ischemia [3,4]. Key anastomoses include the arcade formed by the gastroduodenal and pancreaticoduodenal arteries from the celiac and superior mesenteric arteries, and the watershed area at the splenic flexure formed by the middle colic artery of the superior mesenteric artery and the left colic artery from the inferior mesenteric artery, and the continuous arcade formed by the ileocolic, right, middle, and left colic arteries called the marginal artery of Drummond [3,4]. The arc of Riolan connects the middle colic arteries from the internal iliacs. Further collateralization can evolve in response to vascular compromise [3,4]. 

All three major abdominal arteries originate from the abdominal aorta and receive approximately 20–25% of cardiac output and 30–35% of total blood volume at rest [3,4,5]. Blood is primarily directed toward the mucosa, followed by the muscularis propria and serosa, then the submucosa [3,4,5]. Ischemic injuries are typically confined to the mucosa due to its high metabolic demand and robust blood supply, often recovering once the initial insult resolves [3,4,5]. Prolonged ischemia damages the deeper submucosa and muscularis layers, leading to fibrosis and stricture formation [3,4,5]. Irreversible transmural necrosis and perforation can occur when the ischemia reaches the muscularis and serosa, triggering an inflammatory response that increases vascular permeability and bowel wall breakdown [3,4,5]. Superimposed infection can occur with the translocation of bacteria across these damaged areas [3,4,5]. Studies indicated that the gut can withstand a 75% reduction in blood flow for up to 12 h without significant injury, but complete vascular occlusion can lead to irreversible bowel damage within six hours [6].

AMI encompasses three distinct insult categories that ultimately lead to tissue hypoperfusion [7,8,9]. These insults can include arterial thromboembolisms, mesenteric venous thromboses, or nonocclusive events [7,8,9]. Arterial embolisms resulting from a dislodged thrombus in the left atrium, left ventricle, cardiac valves, or proximal aorta can migrate into the gastrointestinal vasculature and cause an acute occlusion of one of the smaller arteries [7,8,9]. Occasionally, emboli will arise from an atherosclerotic aorta and typically lodge at points of normal anatomic arterial narrowing [10]. The superior mesenteric artery is particularly vulnerable because of its relatively large diameter and low takeoff angle from the aorta [10]. The majority of emboli will lodge 3–10 cm from the origin of the superior mesenteric artery, which spares the proximal jejunum and colon [10]. Acute thrombi may also form in patients with pre-existing chronic mesenteric ischemia from atherosclerosis triggered by abdominal trauma, infection, thrombosed mesenteric aneurysms, and aortic or mesenteric dissections [7,8,9]. Acute arterial occlusions account for approximately 67–95% of AMI cases [7,8,9]. Mesenteric venous stasis in the setting of hypercoagulable states, malignancies, or previous abdominal surgeries can lead to the formation of venous thrombi that can impede forward blood flow through the gut [7,8,9]. Increased resistance to venous blow flow can result in bowel wall edema, promoting ischemic events [7,8,9]. Nonocclusive mesenteric ischemia can also occur in poor systemic or splanchnic circulation or vasoconstriction [7,8,9]. 

### 1.2. Natural History of Acute Mesenteric Ischemia

The clinical presentation of patients with AMI is classically defined as pain disproportionate to the physical exam findings [11]. As the condition progresses, abdominal tenderness may extend over the bowel wall, coinciding with the onset and/or degree of bowel necrosis [11]. The duration and characteristics of pain correlate with the underlying etiology of ischemia with occlusive etiologies demonstrating sudden-onset pain, while non-occlusive causes can be progressive, more generalized, and variable in severity [11]. In some cases, unexplained painless abdominal distension or gastrointestinal bleeding may be the presenting cause in nonocclusive cases, particularly in sedated patients in the intensive care unit [10]. 

The presence of specific comorbidities may elevate the risk for AMI [12]. Older patients with more systemic diseases, such as cardiovascular disease, endocrine and metabolic diseases, renal disorders, vascular disease, and cancer, demonstrate a higher incidence [12]. Hypertension, atherosclerosis, and atrial fibrillation have been identified as predominant comorbidities [13]. In addition, patients with signs of critical illness, such as septic shock, cardiac failure, or respiratory failure, which necessitate the use of vasopressor medications, are also at higher risk [14]. Cases of AMI in patients with COVID-19 have also shown higher rates of AMI due to large vessel thromboembolic events and small-vessel thrombosis related to hypercoagulability and fibrinolysis shutdown [15,16]. AMI-specific mortality rates are notably higher in patients with COVID-19 compared to those without [15,16].

### 1.3. Diagnostic Workflow for AMI

Delayed diagnosis of AMI contributes to its high mortality rate of 30–70% [2]. Bowel ischemia in any critically ill patients should be considered in any unexplained deterioration [10]. The presence of peritoneal signs, such as guarding or rigidity, is an indication for emergent abdominal surgical exploration to assess for bowel perforation, necrosis, and other complications [10]. If an exploratory laparotomy is indicated based on the presence of peritoneal signs, direction palpation of the superior mesenteric artery by placing fingers behind the root of the mesentery can be performed to determine the presence of thrombi [10]. An intraoperative arteriogram may be used and is recommended when the diagnosis is uncertain. Interoperative duplex ultrasonography or flowmetry with fluorescein dye can also be rapidly and reliably employed [10].

High clinical suspicion for AMI in the absence of peritoneal signs should prompt urgent advanced imaging [10]. A biphasic computed tomographic angiography (CTA) protocol is the gold standard diagnostic method due to its high sensitivity and specificity in diagnosing AMI [10]. Pre-contrast scans can detect vascular calcifications, hyper-attenuating intravascular thrombi, and intramural hemorrhages [10]. The following arterial phase with contrast can reveal arterial filling defects within the arteries or regions of infarction within tissues, serving as the initial visible sign of acute mesenteric ischemia [10]. The venous phase may reveal hyper-attenuation within the venous system, concerning the thrombi [10]. Oral contrast should not be used as it can obscure the mesenteric vessels and bowel wall enhancement [10]. Multiplanar reconstructions can help determine the origin of the mesenteric arteries [10]. Blood work is often obtained, including lactate, D-dimer, C-reactive protein, and white blood cell count, but is often nonspecific and does not alter treatment strategies [10].

### 1.4. Treatment Concept Evolution over the Years

Early revascularization of ischemic tissue is vital to improve survival and prevent further bowel loss [10]. Traditionally, AMI has been managed with open surgery due to the ability to concomitantly relieve the arterial occlusion or venous thrombi and evaluate for and resect any necrotic bowel [10]. The advent of minimally invasive endovascular techniques over the past several decades has challenged open revascularization techniques as the gold standard of intervention [10]. Several recent studies have suggested lower mortality rates and rates of bowel resection with newer techniques, but many surgeons and interventionalists still opt for an open approach [10]. Dedicated “intestinal stroke centers” are becoming more prevalent in France and China due to recent evidence suggesting improved outcomes [10]. At these centers, a multidisciplinary team, consisting of a general surgeon, vascular surgeon, interventional radiologist, and intensivist, focuses on removing non-viable small bowel, preserving the remaining intestine with revascularization, and providing intensive care treatment to prevent progression to multiorgan failure [10]. This approach has been associated with improved times to reperfusion and survival [10].

## 2. Methods

**Figure 1:**
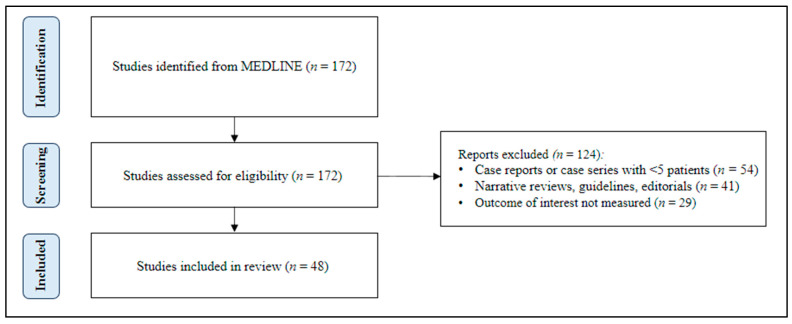
A PRISMA flow diagram of study selection.

To identify relevant studies, a comprehensive search of the Pubmed/MEDLINE database was conducted to retrieve relevant articles from December 2013 to December 2023. A combination of Medical Subject Headings (MeSH) terms and text words related to acute mesenteric ischemia and its treatment strategies were used. MeSH terms included “mesenteric ischemia”, “acute mesenteric ischemia”, “endovascular procedures”, “percutaneous transluminal angioplasty”, “balloon angioplasty”, “open revascularization”, “vascular surgical procedures”, and “stents”. Only the English-language literature involving human subjects was included, and a range of study types, including prospective cohort studies, experimental studies, population studies, meta-analyses, retrospective cohort studies, clinical trials, and observational studies, were eligible for inclusion. After screening and data extraction (Figure 1), the authors conducted a narrative synthesis of the studies, with the extracted data being summarized into tables for easy comparison and review.

## 3. Results

One hundred and seventy-two papers were reviewed, and 48 met the inclusion criteria (Figure 1). Eleven of 48 studies were on open revascularization, 20 were on endovascularization, 13 compared open versus endovascular techniques, and 4 emphasized a hybrid approach. 

## 4. Discussion

### 4.1. Open Revascularization

Optimal treatment for patients with AMI may include an open, endovascular approach, or hybrid approach in a vascular surgery center [17]. Clinical evaluation can raise suspicion of acute peritonitis and, therefore, the need for emergent laparotomy to evaluate the severity of intestinal ischemia [17]. The choice of open surgery technique largely depends on the etiology or location of the occlusion [10]. Embolectomy and angioplasty are well-established definitive treatments for emboli [10]. However, thrombosis located near the aorta–superior mesenteric artery junction may necessitate a bypass procedure [10]. Open techniques allow for direct visualization of the color, dilatation, and peristaltic movement of the intestines to determine the need and extent of resection [17]. When a laparotomy has been performed in patients with peritonitis, exposure of the superior mesenteric artery and balloon embolectomy with a 3 or 4 French catheter through a transverse arteriotomy is indicated [17]. The result is then checked by ultrasonic transit-time flow measurement, but completion angiography of the superior mesenteric artery with anteroposterior and lateral views is optimal [17]. Stenosis and dissection at the arteriotomy closure site, residual peripheral embolus in arterial branches not cleared, and venous return to the portal vein can all be assessed formally [17].

Alternatively, open mesenteric artery bypass can restore blood flow to ischemic tissues if endoluminal salvage is unavailable; however, optimal bypass strategies have not been well studied [18]. A study by Scali et al. in 2019 compared antegrade and retrograde open mesenteric bypass for acute mesenteric ischemia [18]. The cohort comprised 82 patients who underwent aortomesenteric bypass between 2002 and 2016 [18]. Most (76%) underwent antegrade bypass (supraceliac aortic inflow), and the remainder received retrograde infrarenal aortoiliac inflow [18]. Concurrent bowel resection was evenly distributed (antegrade 45%, retrograde 45%; *p* = 0.9), and 37% (*n* = 30) underwent subsequent resection during second look operations [18]. Bypass configuration was not associated with complication rates (*p* > 0.10), in-hospital mortality (log-rank, *p* = 0.3), or overall survival (log-rank, *p* = 0.9) [18]. However, a higher risk of reintervention was observed in patients who underwent retrograde bypass (hazard ratio 3.0, 95% CI 0.9–11.0; *p* = 0.08) [18]. Another study by Huerta et al. in 2020 compared the outcomes of 16 patients who underwent open antegrade bypass to 25 retrograde bypass patients. The retrograde approach was associated with shorter operative times (282 min vs. 375 min; *p* < 0.05), but there were no differences in blood loss, need for second-look laparotomy, morbidity, mortality, length of stay, discharge disposition, or survival [19]. Overall, open mesenteric bypass configuration should be left to the expertise and preference of the surgeon with careful consideration of patient anatomy as the outcomes are similar between the two approaches.

Retrograde open mesenteric stenting has also been studied as an alternative to traditional bypass or embolectomy techniques [20]. A study from 2020 by Andraska et al. compared the outcomes of 16 patients who underwent mesenteric bypass to 18 patients who underwent retrograde open mesenteric stenting at a single institution between 2008 to 2019 [20]. The patients in the stenting group tended to be older and male compared to the bypass group [20]. Overall, the outcomes appeared to be similar at a one-year follow-up [20]. One patient who received a stent was found to have in-stent thrombosis and required mesenteric bypass [20]. One patient who underwent bypass experienced thrombosis in the conduit, leading to perioperative death [20]. Another bypass anastomosis stenosed requiring angioplasty [20]. Complication and mortality rates were otherwise similar [20]. Another study by Gustavo et al. in 2018 identified 29 patients who underwent retrograde open mesenteric stenting for AMI and found the technical success of the procedure to be high (98%) but still demonstrated high early mortality (45%) and morbidity rates (73%) [21]. Other studies by Cirillo-Penn et al. in 2023, Blauw et al. in 2014, Senemaud et al. in 2021, and Roussel et al. in 2018 also corroborate the high technical success of retrograde open mesenteric stenting, but also its high early morbidity and mortality rates and high rates of associated bowel resection [22,23,24,25].

Regardless of the intervention modality, open approaches often occur in a staged approach, commonly called “second-look” [26]. Damage-control surgery is performed to emergently remove any impedance to blood flow and resect necrotic bowel, followed by a “second-look” procedure to remove any residual necrosis [26]. Some surgeons leave the bowel open between procedures, while others prefer a primary fascial closure [26]. A study by Ding et al. in 2017 compared 44 patients who maintained an open abdomen to 65 who had a primary fascial closure after diagnosis with acute superior mesenteric artery occlusion between January 2005 and June 2016 [26]. There was no difference in bowel resection length between the groups in the first emergency surgery [26]. More non-open abdomen patients (35.4%) required an enterectomy during the second-look laparotomy compared to open abdomen patients (13.36%, *p* < 0.05) [26]. A mean of 134 cm of residual alive bowel was present in open abdomen patients compared to 96 cm in non-open abdomen patients [26]. More non-open abdomen patients also suffered from intra-abdominal sepsis (23.1% vs. 6.8%, *p* < 0.01), intra-abdominal hypertension (31% vs. 0, *p* < 0.01), and acute renal failure (53.8% vs. 31.8%, *p* < 0.05) than the open abdomen group after surgery [26]. Short bowel syndrome occurred more infrequently in the open abdomen than in the non-open abdomen group (9.1% vs. 36.9%, *p* < 0.01) [26]. The 30-day (27.3% vs. 52.3%, *p* <0.01) and 1-year (31.8% vs. 61.5%, *p* <0.01) mortality rates were also reduced in the open abdomen group compared to the non-open abdomen group [26].

A study by Swerdlow et al. in 2019 found the overall 30-day mortality rate in 918 patients with AMI who underwent open revascularization to be 32%, specifically 35% after embolectomy, 31% after thromboendarterectomy, and 28% after mesenteric bypass [27]. Mortality was higher in patients who required concomitant bowel resection (38% vs. 29%, respectively; *p* < 0.01) [27]. Disseminated cancer was most strongly associated with 30-day mortality (odds ratio 8.8, 95% CI 2.4–32; *p* = 0.001) [27]. Other factors independently associated with mortality were renal dysfunction, preoperative intubation, preoperative blood transfusion, diabetes, an elevated preoperative international normalized ratio, elevated preoperative white blood cell count, and increasing age [27]. The presence or absence of these factors can aid in preoperative risk stratification [27]. 

A retrospective study identified 32 patients who underwent open thrombectomy for acute superior mesenteric venous thrombosis [28]. Comparisons were made between 17 patients with postoperative systemic anticoagulation and 15 patients with postoperative catheter-directed thrombolysis [28]. The rate of complete thrombus removal was significantly higher in patients who received catheter-directed thrombolysis compared to systemic anticoagulation (80.0% vs. 29.4%, respectively; *p* = 0.001) [28]. Second-look laparotomies and repeat bowel resections were required less frequently in patients who received catheter-directed thrombolysis versus systemic anticoagulation (70.6% vs. 20.0%, *p* = 0.001) [28]. The incidence of short-bowel syndrome (6.7% vs. 41.2%, *p* = 0.001) and 30-day mortality (6.7% vs. 41.2%, *p* = 0.001) were also lower in patients who received catheter-directed thrombolysis [28]. The 1-year survival (93.3% vs. 52.9%, *p* = 0.014) was also better in patients who received catheter-directed thrombolysis [28]. However, the incidence of massive abdominal hemorrhage requiring blood transfusion and surgical intervention was higher in those who received catheter-directed thrombolysis compared to systemic anticoagulation (20% vs. 11.8%, *p* = 0.645), although the difference was not statistically significant [28]. Overall, postoperative catheter-directed thrombolysis may have better clinical outcomes than postoperative systemic anticoagulation.

### 4.2. Endovascular Revascularization

Endovascular revascularization techniques have grown in popularity over the past several decades. Studies have continuously demonstrated their utility as an alternative to open revascularization in the appropriate patient populations. One study by Pengerma et al. in 2023 compared short- and long-term outcomes of endovascularization in 50 elderly patients with AMI compared to no attempted revascularization in 16 [29]. Endovascularization was technically successful in 88% of cases, and three patients underwent subsequent open revascularization after failure [29]. A total of 33% of patients required open resection of necrotic bowel following endovascularization [29]. The 30-day mortality rate was 32% in those who received intervention and 81% in those who did not [29]. One-year survival rates were 52% in the intervention group and 19% for the non-intervention group [29]. Another study by Altintas et al. in 2021 studied the survival rates, rates of bowel resection, complications, re-intervention rate, and improvement of clinical symptoms in 67 patients who underwent endovascular intervention for acute on chronic mesenteric ischemia [30]. One- and three-year survival rates were 67% (95% CI 54–77) and 54% (95% CI 41–65). Only 59% of patients reported clinical improvement in their symptoms [30]. Thirty patients (45%) underwent subsequent bowel resection (*p* < 0.001) with an average length of hospital stay of 7 days [31]. Another study by Karkkainen et al. performed endovascular interventions on 50 patients with AMI secondary to embolic or thrombotic obstruction of the superior mesenteric artery [31]. Endovascular interventions were technically successful for 44 (88%) patients [31]. Mortality after successful or failed endovascular intervention was 32% [31]. The rates of emergency laparotomy, bowel resection, and endovascular intervention-related complications were 40%, 34%, and 10%, respectively [31]. Three of six patients with failure of the endovascular interventions were then treated with surgical bypass [31]. Endovascular intervention failure did not significantly affect survival [31]. Finally, a 12-year retrospective analysis of a single center’s endovascular management of AMI was conducted by Raupach et al. in 2016 [32]. A total of 37 patients underwent primary endovascular intervention with subsequent laparotomy for AMI [32]. Successful recanalization occurred in 91.9% of patients [32]. One patient was successfully treated with surgical embolectomy due to a failed endovascular approach [32]. A subsequent exploratory laparotomy was performed in 73.0% and necrotic bowel resection in 40.5% [32]. The in-hospital mortality was 27.0% [32].

A study by Garzelli et al. in 2022 demonstrated that reperfusion injury could be a frequent complication of endovascular revascularization of AMI, especially in those who present with decreased bowel wall enhancement on pretreatment CT, anabolic cause, and complete occlusion of the superior mesenteric artery [33]. However, it does not seem to impact short-term survival negatively [33].

Overall, endovascularization demonstrated better outcomes than no interventions and could be considered an alternative to open surgery in non-surgical candidates. However, this approach does not mitigate the morbidity and mortality associated with AMI. Subsequent open bowel resection may still be indicated regardless of the initial intervention technique. A retrospective study by Serracant Barrera et al. in 2015 demonstrated an increased survival rate in patients with AMI without peritoneal irritation at diagnosis compared to other interventions [34]. This study shows that endovascular intervention may be useful in early presentations of AMI. 

#### Endovascular Techniques (Aspiration Thrombectomy, Transcatheter Thrombolysis, Stenting)

Endovascularization techniques vary by availability, institution, and skillfulness of interventionalists but can include thrombectomy, stenting, thrombolysis, or a combination of the three. A study by Najdawi et al. in 2022 performed a retro- and prospective analysis of the long-term outcomes of endovascular recanalization by type of intervention performed [35]. A total of 51 out of 58 patients (88%) underwent a successful endovascular intervention, and 10 (17%) experienced a complication [35]. Stenting and in situ thrombolysis occurred in most patients (*n* = 33 and *n* = 19, respectively) [35]. A total of 55% of patients required no further treatment after the procedure, while 16%, 9%, and 9% underwent 2nd-line bowel resection, surgical revascularization, or both [35]. Overall, 79%, 78%, and 63% were alive at 3 months, 1 year, and 3 years [35]. There were no significant differences between intervention techniques [35].

A study by Sun et al. in 2020 evaluated the efficacy and outcomes of transcatheter thrombolysis in 58 patients with acute mesenteric venous thrombosis [36]. Of these patients, 36 did not require any additional intervention; however, 10 developed post-ischemic intestinal stenosis and developed irreversible intestinal ischemia [36]. A total of 24 of the initial cohort also underwent a laparotomy, with 22 patients requiring bowel resection for reversible intestinal ischemia [36]. Statistical analysis determined the presence of leukocytosis above 12 × 10^9^/L (OR = 2.058, 95% CI 1.085–3.903, *p* = 0.027) and an elevated Acute Physiology and Chronic Health Evaluation (APACHE) II score above 8.5 (OR 2.368, 95% CI 1.047–5.357, *p* = 0.038) to be independent predictors of irreversible intestinal ischemia [36]. The overall 30-day mortality rate was 8.6% [36]. Patients with reversible intestinal ischemia demonstrated a higher 30-day mortality and a longer in-hospital stay compared to those patients who did not require resection [36]. These data suggest that transcatheter thrombolysis could be considered a reasonable endovascular technique in patients with early AMI in the absence of irreversible intestinal ischemia [36]. In addition, an elevated APACHE II score and leukocytosis could be used to predict the need for bowel resection, which necessitates a primary open surgical approach [36]. 

Another study by Puippe et al. in 2015 retrospectively analyzed the clinical outcomes of catheter-directed thrombolysis and aspiration thrombectomy in patients with AMI [37]. A total of 13 patients underwent emergent endovascular revascularization of the superior mesenteric artery (12 embolic, 1 thrombotic) [37]. Eleven underwent both catheter-directed thrombolysis and aspiration thrombectomy, while two patients had aspiration thrombectomy alone [37]. The technical success with complete superior mesenteric artery perfusion restoration was achieved in 38.5% (*n* = 5) [37]. Adjunctive angioplasty with or without stenting was required in two patients [36]. Overall, 46.2% clinically improved following the intervention [37]. A total of 38.5% required exploratory laparotomy following the endovascular revascularization attempt, with two patients needing a subsequent colectomy and two needing small bowel resections [37]. Overall, the 30-day mortality rate was 30.8% [37]. Catheter-directed thrombolysis and aspiration thrombectomy may be considered feasible endovascular techniques, but this study demonstrates that many patients will still require open surgery to evaluate for necrosis. 

A retrospective analysis by Freitas et al. in 2018 identified 20 patients from 2011 to 2016 who underwent endovascular mechanical thrombectomy for AMI [38]. Technical success was achieved in all patients with a mean procedural time of 28 ± 17 min [38]. A total of 50% of patients also underwent balloon angioplasty, 25% underwent stent deployment, 20% underwent intraoperative selective thrombolysis, and 10% underwent catheter-assisted aspiration [38]. Following recanalization, 70% of patients required open surgery for intestinal resection [38]. Self-limited small perforations occurred in two patients [38]. No other complications were reported [38]. The 30-day mortality was 40% [38]. This study demonstrated that endovascular thrombectomy is a feasible and reasonably quick procedure with comparable outcomes to other interventions [38]; however, many patients will still require open surgical intervention for intestinal resection. 

Another study by Garzelli et al. in 2022 compared manual thrombectomy in 16 patients versus mechanical thrombectomy in 15 patients and found that both were technically successful (81% vs. 73%, respectively) and associated with partial/complete recanalization in most patients (71% vs. 73%, respectively); however, one thrombectomy technique did not appear to be superior to another [39]. Another study by Shi et al. in 2019 demonstrated that thrombectomy using the Solitaire device with manual thromboaspiration could also be used rapidly and effectively [40].

A small retrospective study by Zhang et al. in 2020 identified five patients who underwent percutaneous mechanical thrombectomy between October 2015 and May 2018 [41]. Thrombectomy was performed on the superior mesenteric artery using a 6F Rotarex catheter (Straub Medical, Wangs, Switzerland) in all five patients [41]. Technical success was 100%, with emboli completely removed in three patients and partially removed in two patients [41]. There were no noted complications [41]. Four of the five patients were smoothly discharged from the hospital after the resolution of symptoms [41]. One patient had persistent symptoms of intestinal ischemia, underwent a small intestinal necrosectomy during exploratory laparotomy, and died 4 months later [41]. Overall, the small study shows that mechanical thrombectomy may be a safe and effective technique in the treatment of AMI [41].

A retrospective study by Pedersoli et al. in 2021 sought to evaluate the effectiveness of primary endovascular stenting for AMI [42]. Forty patients between January 2011 and December 2019 were identified [42]. The 30-day mortality rate was found to be 62.5%, with a median overall survival of patients who survived the first 30 days to be 36 ± 18 months [42]. This study shows that if endovascular revascularization is performed, primary stenting could be performed with adequate survival outcomes [42].

A single-center, retrospective cohort study was conducted by Shi et al. in 2022 to investigate the outcomes of stent thrombectomy combined with aspiration versus aspiration alone in the endovascular management of AMI [43]. A total of 14 patients underwent stent thrombectomy plus aspiration, and 27 underwent aspiration alone [43]. Combination therapy was associated with a higher complete clearance rate (44.4% vs. 78.6%, *p* = 0.04), less adjunctive local thrombolysis (48.1% vs. 14.3%, *p* = 0.03), and a shorter length of hospital stay (10.7 days ± 9.0 vs. 5.7 days ± 4.7, *p* = 0.03), compared to aspiration alone [43]. The estimated survival rates at 1 month, 3 months, 6 months, 1 year, and 2 years were 73.2%, 72.5%, 71.4%, 65.3%, and 59.8%, respectively [43]. No significant difference was found in the survival rate between the groups (log-rank test, *p* = 0.96) [43]. The recurrence rates were 8.3% (1/12) and 4.0% (1/25), respectively [43]. Combination therapy with stent thrombectomy and aspiration should be considered due to improved outcomes versus aspiration alone [43].

Various stents are available: bare metal versus covered, drug-eluting versus not, and self-expandable versus balloon-expandable. The superior stent type is widely debated in the literature with limited existing supporting data. 

A prospective study by Girault et al. published their midterm results in 2021, which compared covered versus bare metal stents in both acute and chronic mesenteric ischemia [44]. Study endpoints included primary patency, primary assisted patency, and secondary patency [44]. A total of 86 patients were included between January 2014 and October 2019 [44]. The clinical presentation was AMI in 42 patients, chronic mesenteric ischemia in 31, and asymptomatic in 13 [44]. The technical success rate was 97% [44]. A total of 96 stents were implanted, including 86 proximal covered stents (Advanta V12, *n* = 73; Lifestream, *n* = 13) [44]. The covered stents’ mean length and diameter were 31.5 ± 6.3 mm and 6.9 ± 0.5 mm, respectively [44]. Additional distal bare metal stents were used in 10 patients (12%) to overcome a kinking (*n* = 9) or a dissection (*n* = 1) downstream of the covered stent [44]. All postoperative deaths occurred in patients with AMI (*n* = 11, 13%) [44]. During a median follow-up of 15.6 months (95% CI 15.6 ± 3.6 months), 12 patients (14%) underwent reinterventions for either stent misplacement (*n* = 3), stent recoil (*n* = 3), stent thrombosis (*n* = 2), de novo stenosis at the distal edge of the covered stent (*n* = 2), or gastric ischemia (*n* = 1) [44]. At 1-year follow-up, overall, the primary patency, primary assisted patency, and secondary patency rates were 83% (95% CI 83% ± 9%), 99% (95% CI 99% ± 3%), and 99% (95% CI 99% ± 3%), respectively [44]. At 2 years, the overall primary patency, primary assisted patency, and secondary patency rates were 76% (95% CI 76% ± 13%), 95% (95% CI 95% ± 8%), and 95% (95% CI 95% ± 8%), respectively [44]. Overall, covered stenting demonstrated a good primary assisted patency rate at 2 years at the price of the significant reintervention rate [44].

Another single-center retrospective study by Zhou et al. in 2019 identified 91 patients who underwent primary mesenteric angioplasty and stenting between 2004 and 2017 and evaluated 2 years of stent patency and the reintervention rate [45]. A total of 113 mesenteric vessels were treated with 20 covered stents and 93 bare metal stents [45]. The primary patency rate was 69% at 2 years for both bare metal and covered stents, with an 83% primary patency rate for covered compared to 65% for bare metal stents (*p* = 0.17) [45]. A total of 27 patients were treated for mesenteric artery in-stent restenosis [45]. Of these, two covered stent patients developed significant restenosis (11%) compared to 25 (34%) bare metal stent patients (*p* = 0.02) [45]. Overall, covered stents demonstrated a superior primary patency rate, although not statistically significant, and had a lower rate of significant restenosis requiring reintervention, at 2 years of follow-up compared to bare metal stents [45].

A retrospective study by Mendes et al. in 2018 aimed to illustrate the feasibility and outcomes of superior mesenteric artery stenting using embolic protection devices in treating AMI and chronic mesenteric ischemia [46]. Superior mesenteric artery stenting was performed in 179 patients of whom 65 had the use of embolic protection devices [46]. Indications for embolic protection devices were severe calcification in 22 patients, acute thrombus in 18, and total occlusion in 16 [46]. Bare metal stents were used in 33 patients, covered stents in 26, and both types in six [46]. Adjunctive therapy included thrombolysis in seven patients, thrombectomy in four, and atherectomy in three [46]. The technical success was 100% [46]. There were no instances of filter retention or arterial trauma due to filter manipulation [46]. Distal embolization was noted in four patients, of whom two had AMI [46]. All large emboli were retrieved using catheter aspiration devices, but one small distal embolus was left untreated with no clinical consequences [46]. Two patients had vessel spasms treated with nitroglycerin [46]. Macroscopic debris was noted in 43 patients and was major in 21 or minor in 22 [46]. Of the patients with AMI, five required exploratory laparotomy, and four had bowel resection [46]. Eight additional patients had early complications, including cardiac complications, brachial hematoma, acute cholecystitis, and acute respiratory distress syndrome [46]. There were two early deaths among those who had AMI [46]. Overall, the use of embolic protection devices appears to be safe and feasible with few complications [46]. This technique could be considered in the treatment of AMI [46].

A retrospective study by Forbrig et al. in 2017 investigated the feasibility and outcomes of primary percutaneous stent revascularization in atherosclerotic AMI [47]. The study included 19 consecutive patients with AMI who underwent this revascularization technique [47]. Technical and clinical success rates were 95% and 53% [47]. Seven patients underwent subsequent laparotomy with bowel resection in four cases [47]. The 30-day mortality rate was 42% [47]. As appreciated in other studies, the need for subsequent laparotomy for bowel resection following a technically successful endovascular intervention remains high [47].

Another study by Bulut et al. in 2017 identified 141 patients who underwent primary mesenteric artery stenting over an 8-year period and analyzed their long-term patency [48]. The median follow-up was 32 months (IQR 20–46) [48]. The primary patency rate at 12 and 60 months was 77.0% and 45.0% [48]. The primary assisted patency rate was 90.3% and 69.8% [48]. Secondary patency was 98.3% and 93.6% [48]. The results of this study show exceptional long-term patency rates of percutaneous stenting techniques [48].

### 4.3. Open Revascularization vs. Endovascular Revascularization

Comparisons of endovascular and open surgery are frequently compared against one another in the current literature, likely due to the relative novelty of endovascular recanalization techniques and their intriguing challenge of the current gold standard treatment for AMI. It is important to note that intervention comparisons should be considered conditional as the stage or degree of ischemia and concomitant comorbidities between open and endovascular cohorts do not always coincide. Because AMI mortality is determined primarily by the stage of development, the scale of necrosis, presence of abdominal sepsis, and, to a lesser extent, the method of revascularization, late-presenting patients likely require more aggressive interventions.

One study by Li et al. in 2014 identified 23,744 patients presenting with AMI from the National Inpatient Sample database from 2005 through 2009 [49]. Of these patients, 679 underwent vascular intervention, 514 underwent open surgery, and 165 underwent endovascular therapy [49]. The proportion of patients undergoing endovascular repair increased from 11.9% in 2005 to 30% in 2009 [49]. Open surgical revascularization demonstrated higher mortality rates (39.3% vs. 24.9%; *p* = 0.01) and longer hospital stays (12.9 vs. 17.1 days; *p* = 0.006) than endovascular interventions [49]. During the study period, 14.4% of patients undergoing endovascular procedures required bowel resection (*p* < 0.001) [49]. Endovascular repair was also less commonly associated with the requirement for total parenteral nutrition support (13.7% vs. 24.4%; *p* = 0.025) [49].

A retrospective study by Branco et al. in 2015 compared open versus endovascular interventions in a total of 439 patients with AMI over 6 years [50]. A total of 27 patients underwent endovascular interventions, 389 underwent open surgery, and 23 underwent a hybrid procedure [50]. Only 16 patients who had endovascular interventions avoided a laparotomy [50]. Endovascular therapy was associated with a 2.5-fold decrease in the risk of death (OR 0.4, 95% CI 0.2–0.9, adjusted *p* = 0.018) [50].

Another study by Eslami et al. in 2015 retrospectively compared the outcomes of 573 patients who had open surgery versus 990 patients who underwent endovascular revascularization [51]. Patients were obtained from the Nationwide Inpatient Sample between the years of 2003 to 2011 [51]. The Elixhauser’s comorbidity index was significantly higher in the endovascular intervention group (mean of 3 ± 0.1 vs. 2.7 ± 0.1, *p* = 0.003) [51]. The mortality rate (21.9% vs. 15.3%, *p* < 0.001) and rates of bowel resection (14.9% vs. 9%, *p* < 0.001) were significantly higher for the open surgery group compared to the endovascular intervention group [51]. These rates remained similar as the study period progressed despite higher endovascular utilization [51].

A small retrospective study by Zhang et al. in 2017 compared the outcomes of 12 patients who underwent open surgical revascularization for AMI to 18 who had endorevascularization between February 2007 and October 2012 [52]. The patients were followed for up to 98 months [52]. The technical success rate of endovascular therapy was 44.4%, and partial success was observed in 55.6% of patients [52]. Laparotomy was required in 33.3% of patients [52]. The 30 mortality was 16.7% [52]. With comparison to open surgical intervention, endovascular therapy was associated with lower rates of laparotomy (33.3% vs. 58.3%, *p* = 0.18), significantly shorter average length of bowel resection (88 ± 44 vs. 253 ± 103 cm, *p* = 0.01), and lower mortality rates (16.7% vs. 33.3%, *p* = 0.68) [52]. 

An observational study by Zettervall et al. in 2017 identified 11,294 revascularizations for AMI from the Nationwide Inpatient Sample and Center for Disease Control and Prevention database from 2000 to 2012 [53]. The use of endovascular interventions increased over this time (0.6–1.8/million, *p* < 0.01); however, concurrent declines of open surgery did not occur (1.8–1.7/million) [53]. Overall, inpatient mortality rates decreased for both endovascular and open interventions. Annual population-based mortality decreased during the study period (12.9–5.3 deaths per million/year, *p* < 0.01) [53].

A systematic review with meta-analysis by El Faragy et al. in 2017 identified a total of 3362 patients from 19 observational studies who underwent endovascular treatment for AMI [54]. The peri-interventional mortality was estimated at 0.245 (95% CI 0.197–0.299), and the estimated need for bowel resection was 0.326 (95% CI 0.229–0.439) [54]. The study also compared 3187 patients who underwent endovascular interventions to 4998 who underwent open surgery for AMI [54]. Endovascular therapy was associated with a lower risk of 30-day mortality (OR 0.45, 95% CI), bowel resection (OR 0.45, 95% CI 0.34–0.59, *p* < 0.00001), and acute renal failure (OR 0.58, 95% CI 0.49–0.68, *p* < 0.00001) [54]. No differences were identified in septic complications or the development of short bowel syndrome [54].

Another systematic review with a meta-analysis by Wang et al. in 2022 sought to compare the clinical outcomes between open surgical, endovascular, and conservative interventions in patients with AMI due to superior mesenteric venous thrombosis [55]. A total of 667 patients from eighteen studies were identified [55]. Endovascular treatment demonstrated a significantly higher efficacy rate than the surgery group (94.8% vs. 75.2%, OR = 4.11, 95% CI 1.67–10.10, *p* < 0.05) [55].

A retrospective study by Erben et al. in 2018 identified 10,381 patients admitted for AMI from 2004 to 2014 using the National Inpatient Sample [56]. A total of 4543 patients underwent endovascular interventions, and 5839 were treated with open surgery [56]. Even though a higher proportion of patients in the endovascular group had a moderate to severe Charlson Comorbidity Index compared to the open group (28% vs. 14%, *p* < 0.0001), the endovascular group was associated with a lower mortality rate (12.3% (97.5% CI, 9.8–14.8%) vs. 33.1% (97.5% CI, 29.9–36.2%); *p* < 0.0001) and a lower mean hospitalization cost ($41,615 (97.5% CI, $38,663–$44,567) vs. $60,286 (97.5% CI, $56,736–$63,836); *p* < 0.0001) [56]. After propensity-adjusted logistic regression analysis, the open group retained a significantly higher mortality rate (OR 3.0; 97.5% CI, 2.2–4.1) and cost (mean, $9196; 97.5% CI, $3797–$14,595) compared to the endovascular intervention group [56]. Patients in the open group also had a significantly higher risk for acute kidney injury (*p* < 0.0001) and discharge to a skilled nursing facility versus home (*p* < 0.0001) [56].

A retrospective study by Andraska et al. in 2022 compared the postoperative major adverse events and 30-day mortality between open and endovascular interventions in patients with AMI from multiple hospitals between 2010 and 2020 [57]. A total of 148 patients were identified, with 28 patients who underwent endovascular interventions [57]. There were no statistically significant differences in postoperative major adverse events between open versus endovascular interventions, but open revascularization was associated with a significantly lower odds of bowel resection (OR 0.23, 95% CI 0.13–0.61) [57]. 

Another study by Rebelo et al. in 2022 identified 44 patients with AMI who underwent open (*n* = 27) or endovascular (*n* = 17) intervention [58]. Endovascular techniques were associated with lower rates of postoperative complications compared to open surgery (64.7% vs. 85.2%, *p* < 0.001), but there was no statistically significant difference in mortality rates (29.6% vs. 29.4%, *p* = 0.028) [58].

Another single-institution retrospective analysis by Li et al. in 2022 compared the clinical outcomes of 14 patients who underwent endovascular therapy for AMI to 27 who had open surgery from March 2013 through August 2021 [59]. Endovascular therapy demonstrated better duration surgery time (median 102 vs. 210 min *p* < 0.001), blood loss (median 20 vs. 200 mL, *p* < 0.001), bowel rest time (median 6 vs. 11 days, *p* = 0.022), ICU time (median 1 vs. 5, *p* = 0.004), and ventilator use (0 vs. 11 h, *p* = 0.011) [59]. There was no difference in bowel necrosis, length of necrotic bowel resected, or in-hospital mortality between the two groups [59].

A retrospective study by Kapalla et al. in 2023 aimed to review the outcomes of AMI treatment with an open or endovascular approach associated with laparotomy and to evaluate the endovascular-first strategy in similar clinical situations [60]. A total of 74 patients treated for AMI between 2007–2021 were included in the study [60]. A total of 61 of these patients were treated with open surgery, and 13 were treated endovascularly with subsequent laparotomy [60]. The total in-hospital mortality was 43% (open surgery 41% vs. endovascular 53.8%, *p* = 0.54) [60]. Independent risk factors for in-hospital mortality were pneumatosis intestinalis (*p* = 0.01), increased lactate concentration (*p* = 0.04), and ischemic intestinal sections (*p* = 0.01) [60]. Congestive heart failure (>New York Heart Association classification II) and atrial fibrillation were related to higher mortality [60]. Overall, the outcomes of endovascular and open surgical interventions appeared similar in this study. 

Another systemic review with meta-analysis by Hou et al. in 2022 retrospectively compared the outcomes of open surgery, endovascularization, and retrograde open mesenteric stenting [61]. A total of 2369 patients from 39 studies were included [61]. The pooled mortality estimates tended to be similar over the past 20 years at 40% (95% CI 0.33–0.47, I^2^ = 84%) for open surgery, 26% (95% CI 0.19–0.33; I^2^ = 33%) for endovascular therapy, and 32% (95% CI 0.21–0.44; I^2^ = 26%) for retrograde open mesenteric stenting [61].

### 4.4. Hybrid Approaches to Acute Mesenteric Ischemia

Hybrid approaches to AMI at dedicated intestinal stroke centers have gained popularity over the last decade as they have demonstrated superior outcomes compared to traditional open or endovascular approaches. 

Salsano et al. developed a systematic review with a meta-analysis in 2018 aimed to estimate the prognostic impact of surgical management versus endovascular or hybrid interventions as first-line treatment for AMI and to determine if endovascular strategies are effective in reducing the rates of bowel resection [62]. A total of 3020 patients from seven studies comparing endovascularization versus open surgery were included [62]. Endovascular approaches were associated with a reduced risk of in-hospital mortality (RR 0.68; 95% CI 0.59–0.79; fixed-effects analysis; *p* < 0.0001; I^2^ = 4.9%; τ^2^ = 0.025) [62]. The pooled prevalence of mortality was 19% for endovascularization and 34% for open surgery [62]. Endovascularization positively impacted the risk of bowel resection and second-look laparotomy [62]. The authors concluded that a multidisciplinary approach prioritizing endovascularization prior to open surgery improves the outcomes associated with AMI [62]. 

The evidence of a hybrid intervention strategy of endovascularization combined with open bowel resection has also shown promising outcomes in patient venous thrombosis etiologies of AMI [63]. A study by Liu et al. in 2020 retrospectively analyzed 68 cases of patients who were treated for acute mesenteric venous thrombosis between January 2009 and December 2014 [63]. All 68 cases received systemic anticoagulation, 24 received endovascular intervention, 19 received open surgery, and 25 received endovascular intervention plus bowel resection [63]. The overall mortality rate was 2.94% [63]. Bowel resection rates significantly decreased in the combination therapy group compared to the open surgery group (92 ± 14 cm vs. 162 ± 27 cm, t = −2.377, *p* = 0.022) [63]. At the 1-year follow-up, there were only four cases of short bowel syndrome [63].

A prospective study by Roussel et al. in 2015 corroborated the benefit of intestinal stroke centers [64]. They developed a multimodal and multidisciplinary management strategy for AMI that focuses on intestinal viability and involves gastroenterologists, vascular and abdominal surgeons, radiologists, and critical care specialists [64]. All patients with AMI receive a specific medical protocol, endovascular and/or open surgical revascularization when possible, and/or resection of nonviable intestinal tissue [64]. The study prospectively enrolled 83 patients with AMI, of whom 29 underwent revascularization [64]. Overall, the 2-year survival was 89.2%, and 30-day operative mortality was 6.9% [64]. Surgical revascularization included bypass grafting (65%), endarterectomy with patch angioplasty (21%) ± retrograde open mesenteric stenting of the superior mesenteric artery (7%), and endovascular revascularization as a first stage procedure (38%) [64]. The 2-year primary patency rate of open revascularization was 88% [64]. The rate and the median length of bowel resected were 24% and 43 cm (range, 36–49 cm), respectively [64]. Overall, the outcomes associated with the intestinal stroke center model have demonstrated promising results [64].

Finally, the results of another prospective study by Zientara et al. from 2021 also support the use of an interdisciplinary treatment approach to AMI [65]. The authors prospectively collected and retrospectively evaluated 26 consecutive patients with acute or chronic mesenteric ischemia treated by an interdisciplinary team [65]. During the initial evaluation, the visceral surgeon determined the extent of bowel resection, and the vascular surgeon determined the appropriate method of revascularization [65]. Routine follow-up consisted of clinical examination and ultrasound- or CT-imaging for patency assessment and overall survival [65]. Eighteen of 26 patients underwent open surgical intervention, with ten receiving an iliac-mesenteric bypass [65]. Seven underwent thromboembolectomy of the mesenteric artery [65]. One patient received an infra-diaphragmatic aorto-celiac-mesenteric bypass [65]. Of the eight patients who were not open revascularization candidates, two patients were treated endovascularly, and six underwent explorative laparotomy [65]. The overall in-hospital mortality was 23% [65]. The mean survival of the revascularized group (*n* = 20) was 51.8 months (95% CI 39.1–64.5) compared to 15.7 months in the non-revascularized group (*n* = 6) (95% CI—4.8–36.1; *p* = 0.08) [65]. The median follow-up was 64.6 months [65]. The primary patency in the 16 patients after open and two after interventional revascularization was 100% and 89.9% in the follow-up [65].

## 5. Conclusions and Future Direction

Even with modern advancements in the management of acute mesenteric ischemia, morbidity and mortality remain high, and the best primary treatment modality is still debated amongst interventionalists. Recent data have demonstrated that interventionalists tend to trend more toward endovascular approaches over open surgery, likely due to the apparent reduction in mortality and perioperative complications. Even though the technical and clinical success rates are high, a need for subsequent exploratory laparotomy for the resection of necrotic bowel often remains in a large proportion of patients who undergo endovascular revascularization. This may be explained by the tendency of patients with higher rates of comorbidities to undergo endovascular repair due to their lack of surgical candidacy. Differences in mortality rate may also be explained by the stage of acute mesenteric ischemia at presentation, as primary open surgical approaches are still preferred for patients with any signs of peritonitis upon clinical examination. Data also suggest that hybrid approaches involving primary endovascular interventions with subsequent open laparotomies for bowel resections may be superior to either alone. Intestinal stroke centers create a multimodal, multidisciplinary approach promoting these hybrid interventions and have demonstrated exceptional outcomes. 

Further studies are needed to continue the pursuit of the optimal treatment strategy for AMI. More research should be conducted involving intestinal stroke centers to determine appropriate protocols, roles or interventionalists, and acceptable outcomes that can be universally applied across institutions. Other research areas could focus on comparing specific endovascular techniques, such as embolectomy, stenting, thrombolysis, or combinations of the methods. Drug-coated balloons are novel interventional devices that have been used for coronary artery or peripheral artery stenosis, but studies involving mesenteric arteries are lacking [66,67]. Furthermore, the types of stents used for the revascularization of mesenteric arteries are debated and need more research to determine which is superior. The use of drug-eluting stents in AMI also could benefit from more studies, as there has not been one study on the topic during the past decade. 

As the landscape of revascularization techniques evolves with the release of superior techniques, interventionalists are challenged to stay current and skillful with novel strategies. The institutional availability of educational tools and simulation resources may limit an interventionalist from mastering new crafts. The rarity of AMI, especially in smaller hospital systems, may contribute to an institutional lack of currency. Intestinal stroke centers that urge hybrid approaches may also not be fiscally or practically feasible for more remote or smaller hospital systems. This review invites interventionalists to seek out modernity and challenge themselves with new and improved revascularization strategies to hopefully decrease the morbidity and mortality of the highly lethal condition that is AMI.
